# ISG20 serves as a potential biomarker and drives tumor progression in clear cell renal cell carcinoma

**DOI:** 10.18632/aging.102714

**Published:** 2020-01-30

**Authors:** Tianbo Xu, Hailong Ruan, Su Gao, Jingchong Liu, Yuenan Liu, Zhengshuai Song, Qi Cao, Keshan Wang, Lin Bao, Di Liu, Junwei Tong, Jian Shi, Huageng Liang, Hongmei Yang, Ke Chen, Xiaoping Zhang

**Affiliations:** 1 Department of Urology, Union Hospital, Tongji Medical College, Huazhong University of Science and Technology, Wuhan 430022, China; 2 Department of Geriatrics, Union Hospital, Tongji Medical College, Huazhong University of Science and Technology, Wuhan 430022, China; 3 Department of Urology, The Central Hospital of Wuhan, Tongji Medical College, Huazhong University of Science and Technology, Wuhan 430022, China; 4 Department of Pathogenic Biology, School of Basic Medicine, Huazhong University of Science and Technology, Wuhan 430030, China

**Keywords:** clear cell renal cell carcinoma, ISG20, biomarker, tumor progression, bioinformatics

## Abstract

Clear cell renal cell carcinoma (ccRCC) is one of the most common malignancies and lacks reliable biomarkers for diagnosis and prognosis, which results in high incidence and mortality rates of ccRCC. In this study, ISG20, HJURP, and FOXM1 were identified as hub genes via weighted gene co-expression network analysis (WGCNA) and Cox regression analysis. Samples validation showed that only ISG20 was up-regulated in ccRCC. Therefore, ISG20 was selected for further study. High ISG20 expression was associated with poor overall survival and disease-free survival. Furthermore, the expression of ISG20 could effectively differentiate ccRCC from normal tissues and was positively correlated to clinical stages. Functional experiments proved that knockdown of ISG20 expression could obviously inhibit cell growth, migration, and invasion in ccRCC cells. To find the potential mechanisms of ISG20, gene set enrichment analysis (GSEA) was performed and revealed that high expression of ISG20 was significantly involved in metastasis and cell cycle pathways. In addition, we found that ISG20 could regulate the expression of MMP9 and CCND1. In conclusion, these findings suggested that ISG20 promoted cell proliferation and metastasis via regulating MMP9/CCND1 expression and might serve as a potential biomarker and therapeutic target in ccRCC.

## INTRODUCTION

Renal cell carcinoma (RCC) is one of the common lethal tumors in the urologic system, which is characterized by high incidence and high mortality rates. RCC accounts for 80-90% of all renal tumors [1, 2]. Recent cancer statistics estimated that there will be 73,820 new cases of RCC and 14,770 people will die of RCC in the USA in 2019 [3]. Clear cell renal cell carcinoma (ccRCC) is the most common subtype of RCC, which accounts for approximately 80% of RCC [4–6]. Because of resistance to radiotherapy and traditional chemotherapy, surgery becomes the most effective treatment for localized RCC patients [7]. For advanced RCC or metastatic RCC (mRCC), molecular targeted therapy has become a new first-line treatment [8–11] and promoted the median survival time for approximately 3 years [12]. Unfortunately, many patients are still insensitive to targeted therapy. Therefore, it is necessary to find new biomarkers and therapeutic targets in ccRCC.

Nowadays, microarray [13] and high throughput sequencing [14, 15] techniques are frequently applied to identify generally genetic alterations. Integrated bioinformatics analyses are further used to find potential molecular mechanisms of tumorigenesis and progression. In this study, weighted gene co-expression network analysis (WGCNA) and cox regression analysis were utilized to screen hub genes in ccRCC.

Previous studies revealed that the interferon stimulated genes (ISGs) produce proteins acted as antiviral effectors in many virus infectious diseases [16–18]. Interferon stimulated exonuclease Gene 20 (ISG20), also named as estrogen-regulated transcript 45 protein, is an RNA exonuclease which induced by interferons (IFN types I and II) or double-stranded RNA [19–21]. ISG20 is able to cleave single-stranded RNA or DNA and is significantly associated with host antiviral innate immune defense [22, 23]. Furthermore, previous studies indicated that ISG20 played a vital role in tumorigenesis and progression of neoplasms. ISG20 with exonuclease activity could promote angiogenesis in vitro [24]. Lin et al. also demonstrated that ISG20 enhanced angiogenesis and supported progression of hepatocellular carcinoma (HCC) regulated by thyroid hormone [25]. To the best of our knowledge, the function of ISG20 has not been reported in ccRCC. In this study, we focused on the biological function and molecular mechanism of ISG20 via integrated bioinformatics analysis and functional experiments of ccRCC in vitro.

## RESULTS

### Identification of Differentially Expressed Genes (DEGs) in ccRCC

**Figure 1:**
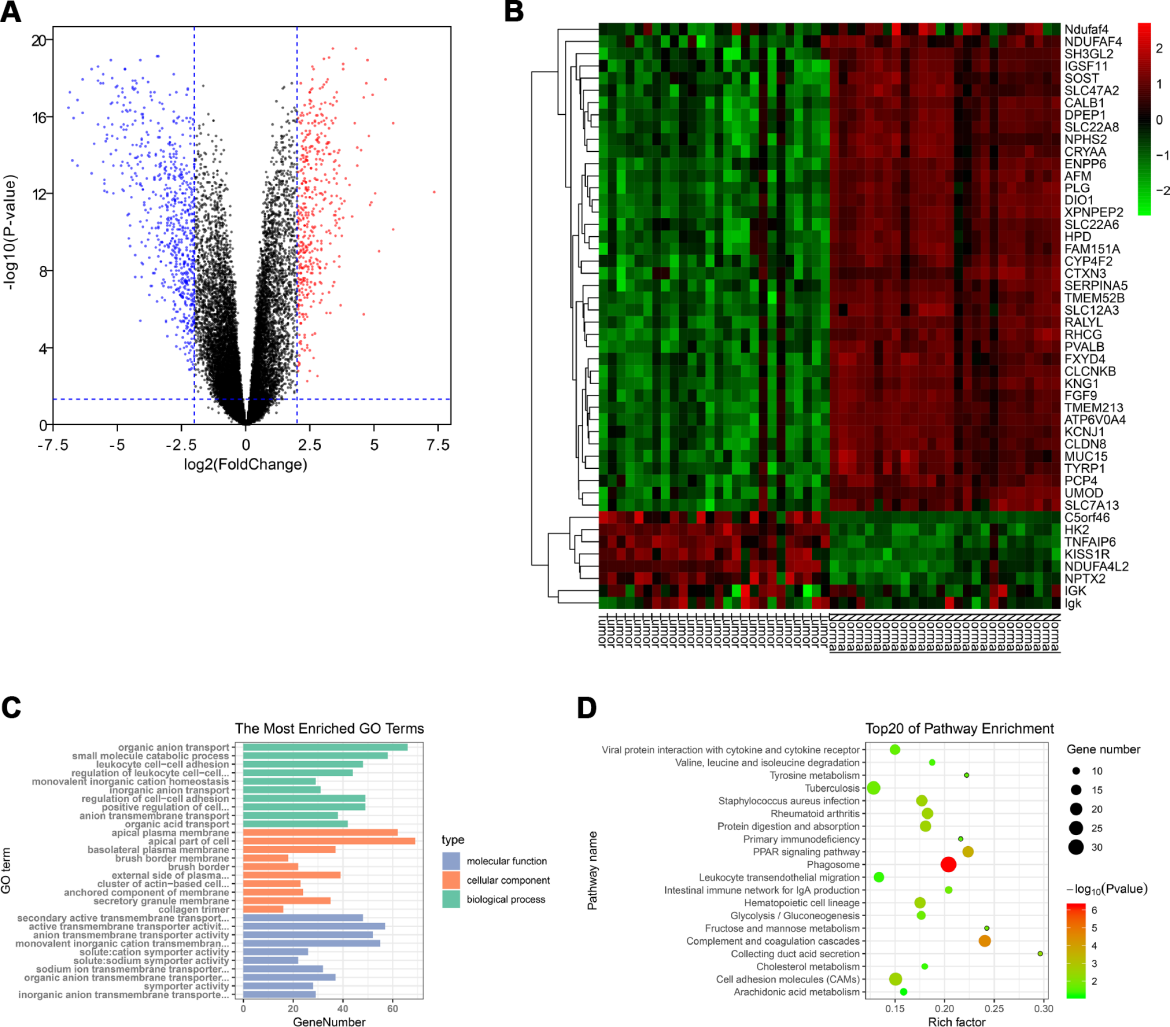
**Identification of DEGs and functional annotation.** (**A**) The DEGs were exhibited as a volcano plot. Red dot: up-regulated genes; Blue dot: down-regulated genes; Black dot: unchanged genes. (**B**) Heatmap of the top 50 genes. (**C**) GO enrichment analysis of DEGs. Green columns: biological process group; Orange columns: cellular component group; Blue columns: molecular function group. (**D**) KEGG pathway enrichment analysis of DEGs. Top 20 enriched pathways were exhibited. DEGs: differentially expressed genes; GO: Gene Ontology; KEGG: Kyoto Encyclopedia of Genes and Genomes.

Gene expression data and clinical data were extracted from the GSE66272 dataset. According to the cut-off criteria, a total of 1025 genes were identified as DEGs (Figure 1A). In addition, the top 50 genes were exhibited in a heat map (Figure 1B).

### Functional enrichment analysis of DEGs

The “clusterProfiler” package was used to perform GO and KEGG enrichment analysis in R. As shown in Figure 1C, biological process analysis indicated that the DEGs were significantly associated with organic anion transport, small molecule catabolic process, and leukocyte cell-cell adhesion. Cellular component results revealed that the DEGs mainly located in the apical plasma membrane, apical part of cell and basolateral plasma membrane. In the molecular function group, the DEGs were obviously enriched in secondary active transmembrane transporter activity, active transmembrane transporter activity, and anion transmembrane transporter activity. Moreover, the KEGG pathway enrichment analysis was performed to further uncover the potential biological functions of DEGs. As shown in Figure 1D, KEGG analysis exhibited that the DEGs were significantly correlated to the PPAR signaling pathway, cell adhesion molecules and multiple metabolic pathways (fructose/mannose metabolism, glycolysis/gluconeogenesis, tyrosine metabolism, cholesterol metabolism and arachidonic acid metabolism).

### Screening hub genes via WGCNA and Cox regression analysis

**Figure 2:**
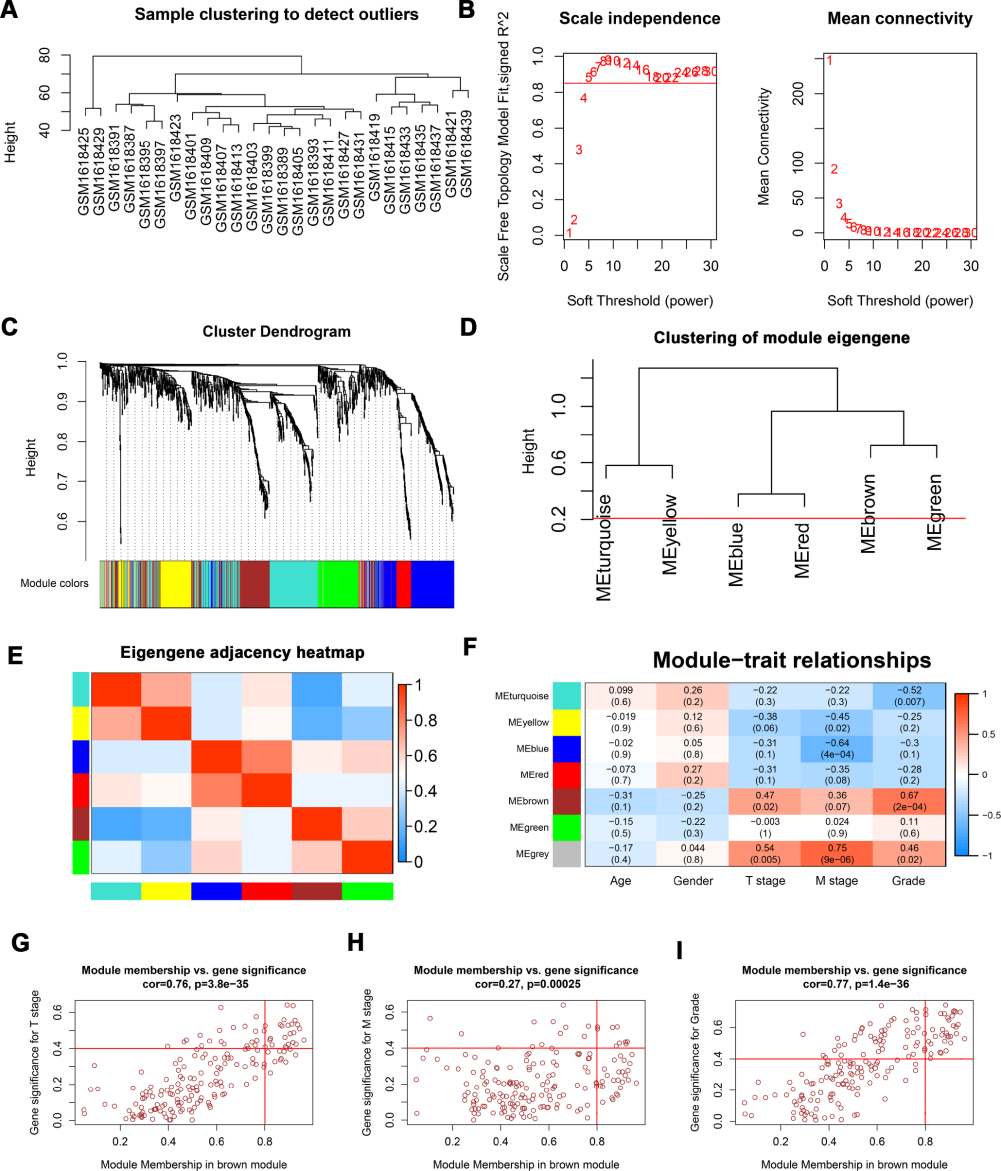
**WGCNA of DEGs.** (**A**) Sample clustering of GSE66272 to detect outliers. (**B**) Analysis of scale-free fit parameter and mean connectivity for various soft-thresholding power. (**C**) Dendrogram of DEGs clustered based on a dissimilarity measure (1-TOM). The DEGs were divided into seven modules (turquoise, yellow, blue, red, brown, green and grey module). (**D**, **E**) Clustering of module eigengene and eigengene adjacency heatmap to calculate the correlation between each module. No modules need to be merged. (**F**) The brown module was selected as the most important module according to the correlation between MEs and clinical traits. (**G**–**I**) Nine genes (TOP2A, NUF2, KIF4A, HJURP, FOXM1, CDCA8, CDCA5, CDC45, and ISG20) were identified as key genes in the brown module due to significantly associated with T stage, M stage, and Grade. WGCNA: weighted gene co-expression network analysis; DEGs: differentially expressed genes; TOM: topological overlap matrix; MEs: module eigengenes; MM: module membership; GS: gene significance.

**Figure 3:**
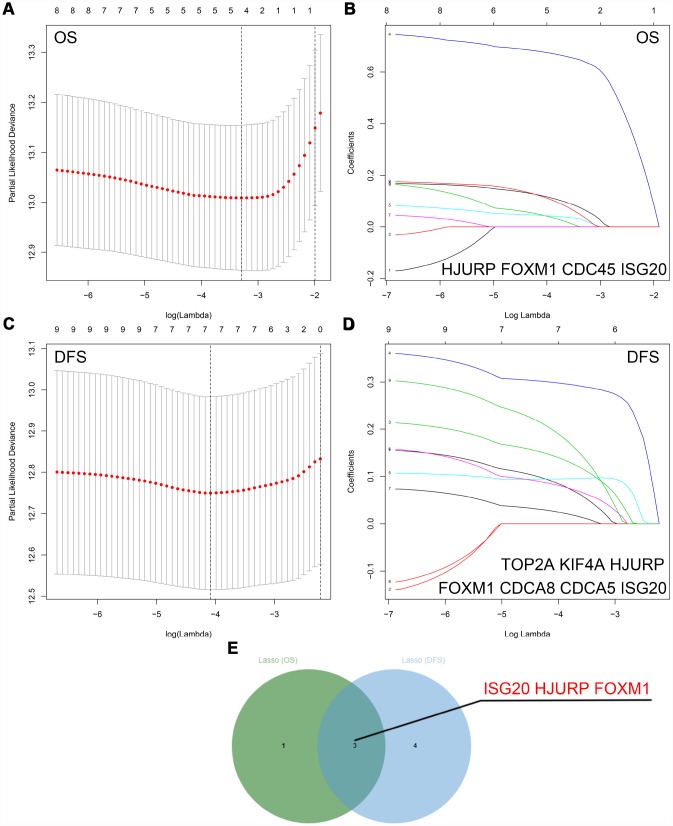
**Identification of hub genes via LASSO cox regression analysis.** (**A**) Partial likelihood deviance of OS for the LASSO coefficient profiles. (**B**) LASSO coefficient profiles of the nine key genes for OS. (**C**) Partial likelihood deviance of DFS for the LASSO coefficient profiles. (**D**) LASSO coefficient profiles of the nine key genes for DFS. (**E**) ISG20, HJURP, and FOXM1 were identified as hub genes according to the LASSO cox regression analysis of OS and DFS. LASSO: least absolute shrinkage and selection operator; OS: overall survival; DFS: disease-free survival.

The “WGCNA” package was utilized to screen hub modules significantly related to clinical characters. In our study, the power of β = 5 was selected as the soft threshold to ensure a scale-free network (Figure 2B). As shown in Figure 2C, seven modules (turquoise, yellow, blue, red, brown, green and grey module) were identified based on the gene expression pattern. Furthermore, the correlations between modules were all less than 0.8 (Figure 2D, 2E) and no modules need to be merged. According to the correlations between modules and clinical traits, the brown module (T stage: r = 0.47, p = 0.02; M stage: r = 0.36, p = 0.07; Grade: r = 0.67, p = 2e-04) was identified as a hub module for further analysis (Figure 2F). In this study, we selected T stage, M stage, and Grade as cutoff parameters to screen key genes (Figure 2G, 2I). Nine genes (TOP2A, NUF2, KIF4A, HJURP, FOXM1, CDCA8, CDCA5, CDC45, and ISG20) with significant clinical correlation were identified as key genes in the brown module (Table 1). Then, univariate and LASSO cox regression analysis of OS and DFS were performed to further screen hub genes. Univariate cox regression analysis indicated that nine key genes regarded as prognostic factors in both OS and DFS (Table 2). In LASSO cox regression, four genes (HJURP, FOXM1, CDC45, and ISG20) were identified in OS analysis (Figure 3A, 3B) and seven genes (TOP2A, KIF4A, HJURP, FOXM1, CDCA8, CDC45 and ISG20) were identified in DFS analysis (Figure 3C, 3D). As shown in Figure 3E, ISG20, HJURP and FOXM1 were identified as hub genes according to the results of cox regression analysis.

**Table 1:** Key genes identified by WGCNA.

**Gene**	**Cor.geneModuleMembership**		**Cor.geneTraitSignificance**		
	**ME brown**		**T stage**	**M stage**	**Grade**
TOP2A	0.920		0.487	0.423	0.706
NUF2	0.864		0.532	0.514	0.689
KIF4A	0.893		0.640	0.429	0.662
HJURP	0.879		0.559	0.411	0.688
FOXM1	0.917		0.639	0.434	0.701
CDCA8	0.884		0.462	0.518	0.743
CDCA5	0.925		0.556	0.436	0.699
CDC45	0.802		0.525	0.506	0.682
ISG20	0.904		0.483	0.461	0.715

WGCNA: weighted gene co-expression network analysis; ME: module eigengene.

**Table 2:** Univariate cox regression analysis of key genes.

**Variable**	**Univariate analysis (OS)**				**Univariate analysis (DFS)**		
	**HR**	**95%CI**	**P-value**		**HR**	**95%CI**	**P-value**
TOP2A	1.774	1.305-2.413	0.000		1.906	1.321-2.749	0.001
NUF2	1.840	1.353-2.503	0.000		1.673	1.169-2.394	0.005
KIF4A	1.984	1.456-2.702	0.000		1.942	1.350-2.794	0.000
HJURP	2.594	1.879-3.579	0.000		2.138	1.487-3.075	0.000
FOXM1	2.118	1.550-2.894	0.000		2.026	1.407-2.917	0.000
CDCA8	2.052	1.500-2.806	0.000		1.954	1.359-2.810	0.000
CDCA5	2.027	1.482-2.771	0.000		1.896	1.320-2.725	0.001
CDC45	1.894	1.395-2.573	0.000		1.530	1.071-2.185	0.019
ISG20	1.652	1.219-2.239	0.001		1.703	1.188-2.441	0.004
Age	1.750	1.288-2.378	0.000		1.363	0.957-1.942	0.086
Gender	0.942	0.692-1.282	0.704		1.427	0.960-2.120	0.079
T stage	1.901	1.615-2.237	0.000		2.506	2.033-3.089	0.000
N stage	3.847	2.083-7.107	0.000		5.731	2.874-11.428	0.000
M stage	4.295	3.149-5.856	0.000		8.628	5.944-12.524	0.000
TNM stage	1.875	1.645-2.138	0.000		2.668	2.238-3.180	0.000
Grade	2.250	1.842-2.748	0.000		3.028	2.348-3.905

OS: overall survival; DFS: disease-free survival; HR: hazard ratio; CI: confidence interval; TNM: Tumor Node Metastasis.

### Hub genes validation

**Figure 4:**
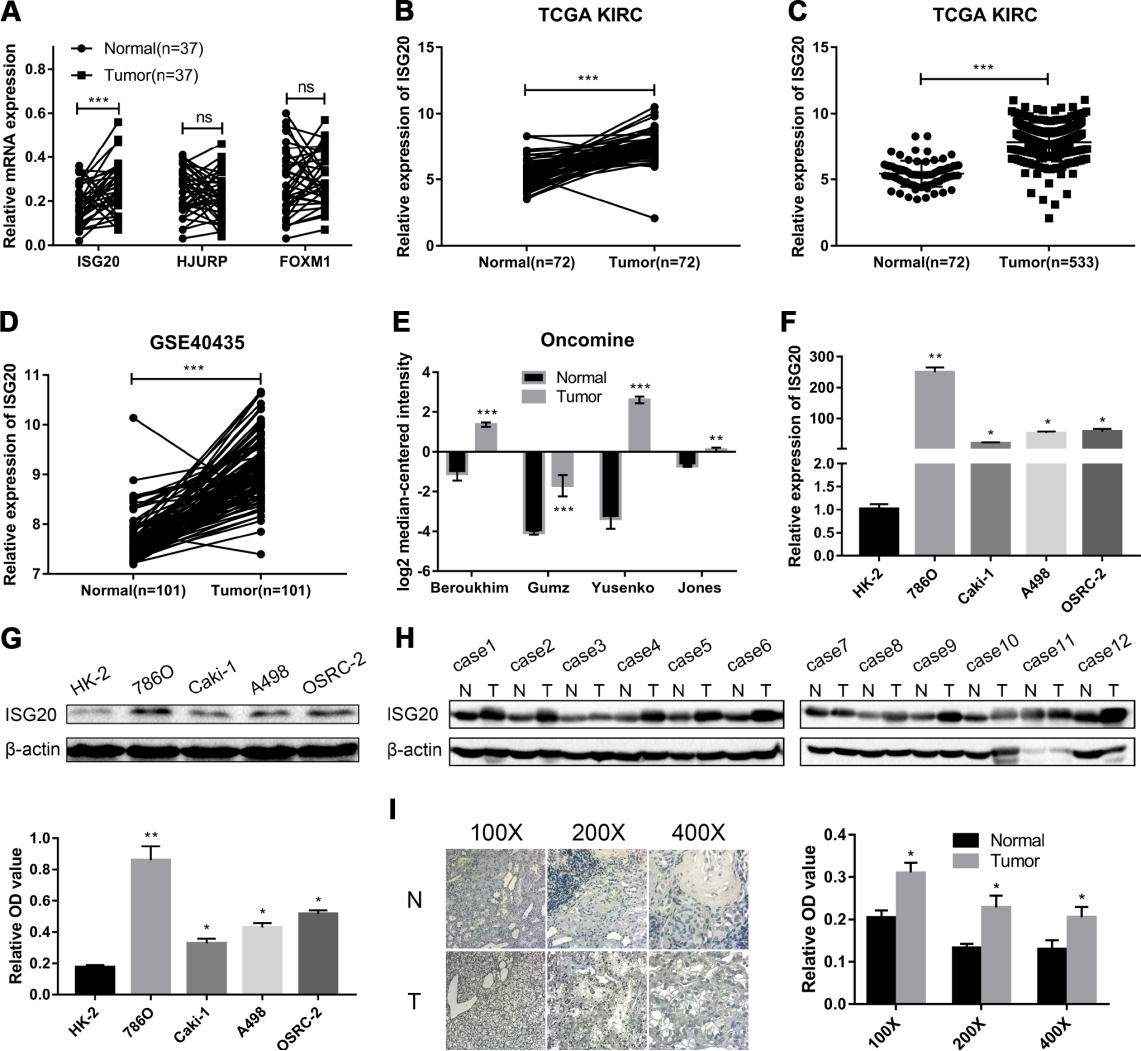
**ISG20 was up-regulated in ccRCC.** (**A**) The mRNA expression level of ISG20 was elevated in ccRCC tissues. (**B**–**E**) ISG20 was up-regulated in ccRCC tissues in the online databases (TCGA KIRC dataset, GSE40435 dataset, and Oncomine database). (**F**, **G**) Both mRNA and protein expression levels of ISG20 were elevated in ccRCC cell lines. (**H**, **I**) The protein expression level of ISG20 was higher in ccRCC tissues than that in normal tissues. ccRCC: clear cell renal cell carcinoma; TCGA KIRC: The Cancer Genome Atlas Kidney Clear Cell Carcinoma; qRT-PCR: quantitative real-time PCR; IHC: Immunohistochemistry. Data are represented as mean ± SD. ***, P < 0.001; **, P < 0.01; *, P < 0.05.

RT-PCR assay was performed to verify the mRNA expression level of hub genes in ccRCC tissues. We selected ISG20 for further study as only ISG20 was elevated in ccRCC tissues (Figure 4A). Then, we further validated the expression level of ISG20 in other databases. In the TCGA KIRC and GSE40453 datasets, the expression of ISG20 in the ccRCC group was significantly higher than that in the control group (Figure 4B–4D). In the Oncomine database, ISG20 was also obviously up-regulated in ccRCC tissues (Figure 4E). Moreover, we found that ISG20 was elevated both in mRNA and protein levels of ccRCC cell lines (Figure 4F, 4G). And as shown in Figure 4H–4I, the protein expression level of ISG20 was also up-regulated in ccRCC tissues. Therefore, we believe that ISG20 is indeed up-regulated in ccRCC.

### The expression level of ISG20 was significantly associated with clinicopathological features

**Figure 5:**
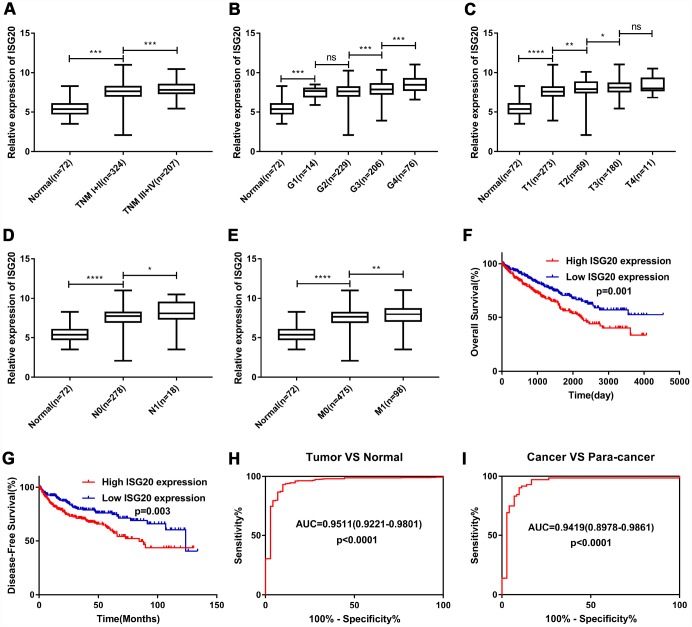
**ISG20 expression level was associated with various clinicopathological parameters in ccRCC.** The expression of ISG20 was positively correlated to TNM stage (**A**) Grade (**B**) T stage (**C**) N stage (**D**) and M stage (**E**). High expression of ISG20 predicted poor OS (**F**) and DFS (**G**) in ccRCC. (**H**, **I**) The expression of ISG20 could effectively differentiate ccRCC from normal renal tissues. ccRCC: clear cell renal cell carcinoma; TNM: Tumor Node Metastasis; OS: overall survival; DFS: disease-free survival. Data are represented as mean ± SD. ****, P < 0.0001; ***, P < 0.001; **, P < 0.01; *, P < 0.05.

As shown in Figure 5A–5E, the expression of ISG20 was observably positively correlated with multiple clinical stages (TNM stage, Grade stage, T stage, N stage and M stage). The survival analysis (Figure 5F–5G) showed that high expression of ISG20 predicted poor OS (HR = 1.64, p = 0.001) and DFS (HR = 1.70, p = 0.003). In addition, ROC curve analysis also exhibited that ISG20 had good diagnostic value in ccRCC. The expression level of ISG20 could effectively differentiate ccRCC from normal renal tissues (Figure 5H, 5I). Thus, ISG20 may become a potential diagnostic and prognostic biomarker in ccRCC.

### ISG20 promoted cell proliferation, invasion, and migration in ccRCC

**Figure 6:**
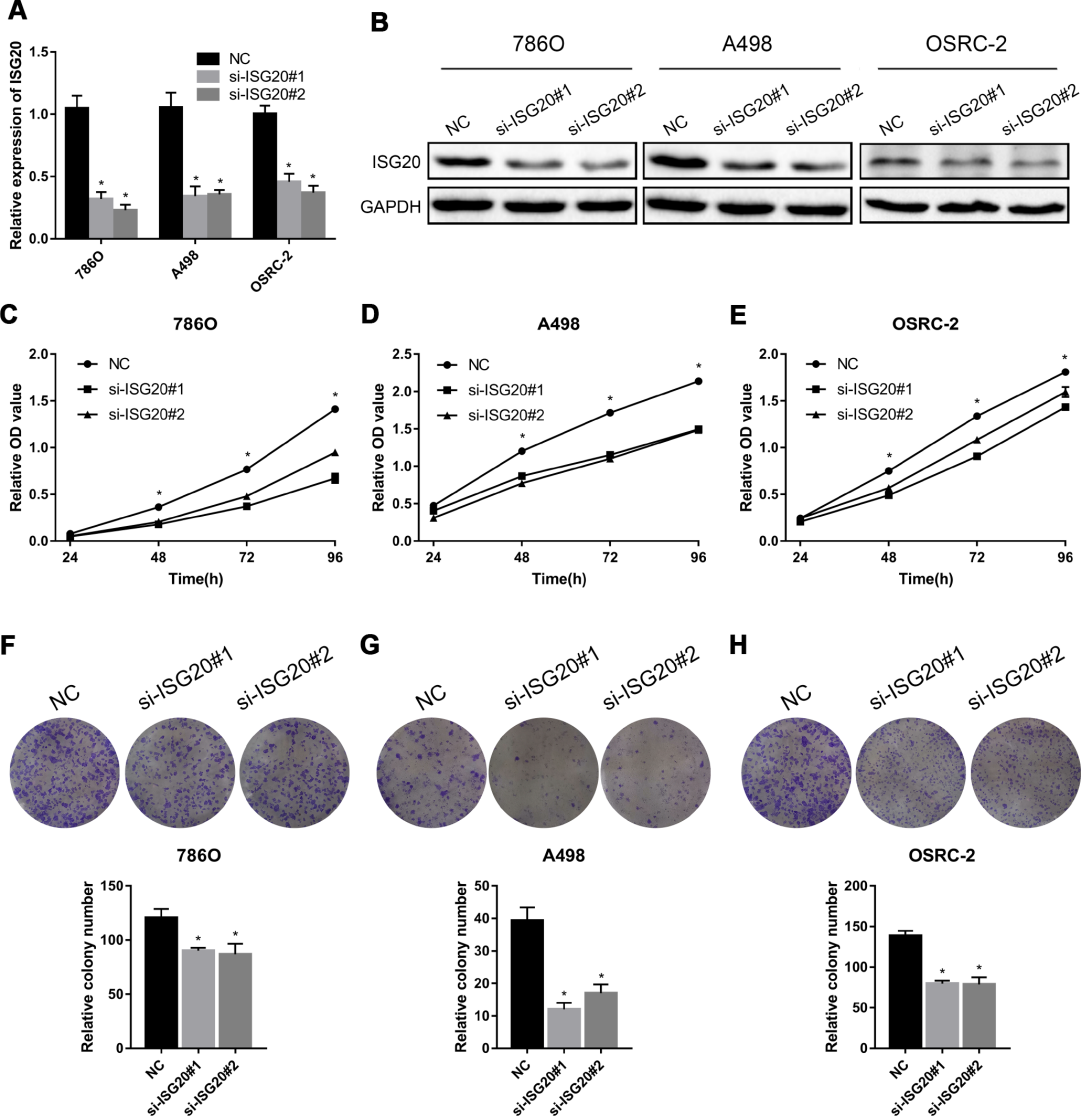
**ISG20 promoted ccRCC cell proliferation in vitro.** (**A**-**B**) The si-ISG20 could effectively inhibit the expression of ISG20. (**C**–**E**) CCK-8 assays were used to detect the effect of ISG20 knockdown on the proliferation of ccRCC cell lines (786O, A498, and OSRC-2). (**F**–**H**) Clone formation assays were used to detect the effect of the ISG20 knockdown on the clone formation ability. ccRCC: clear cell renal cell carcinoma; CCK-8: cell counting kit - 8. Data are represented as mean ± SD. ***, P < 0.001; **, P < 0.01; *, P < 0.05.

**Figure 7:**
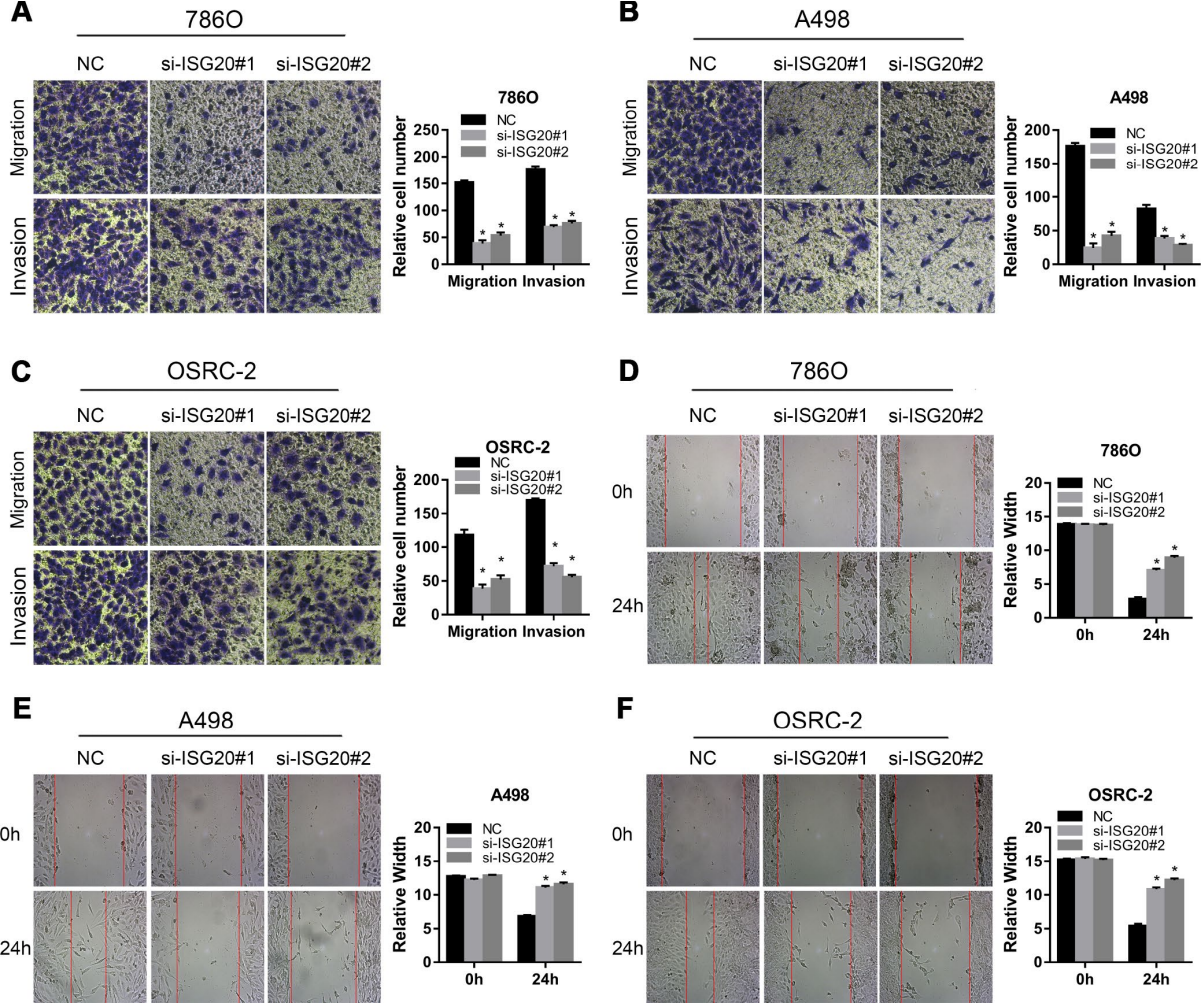
**ISG20 improved the migration and invasion abilities of ccRCC cells in vitro.** (**A**–**C**) Transwell assays analysis of the effect of ISG20 knockdown on cell migration and invasion. (**D**–**F**) Wound healing assays analysis of the effect of ISG20 knockdown on the migration of ccRCC cells. ccRCC: clear cell renal cell carcinoma. Data are represented as mean ± SD. ***, P < 0.001; **, P < 0.01; *, P < 0.05.

To verify the effect of ISG20 on the biological function of ccRCC cell, the siRNA targeting ISG20 (si-ISG20) was used to knockdown the expression of ISG20 in 786O, A498, and OSRC-2 cell lines. The RT-PCR and western blotting results indicated that the si-ISG20 could effectively inhibit the expression of ISG20 in ccRCC cell (Figure 6A, 6B). As shown in Figure 6C–6H, down-regulation of ISG20 significantly inhibited cell proliferation. Furthermore, down-regulation of ISG20 also dramatically reduced the capacity of invasion and migration in ccRCC cells (Figure 7). These findings suggested that ISG20 drove tumor progression and played the role of the oncogene.

### ISG20 facilitated proliferation and metastasis of ccRCC via mediating the expression of CCND1/MMP9

**Figure 8:**
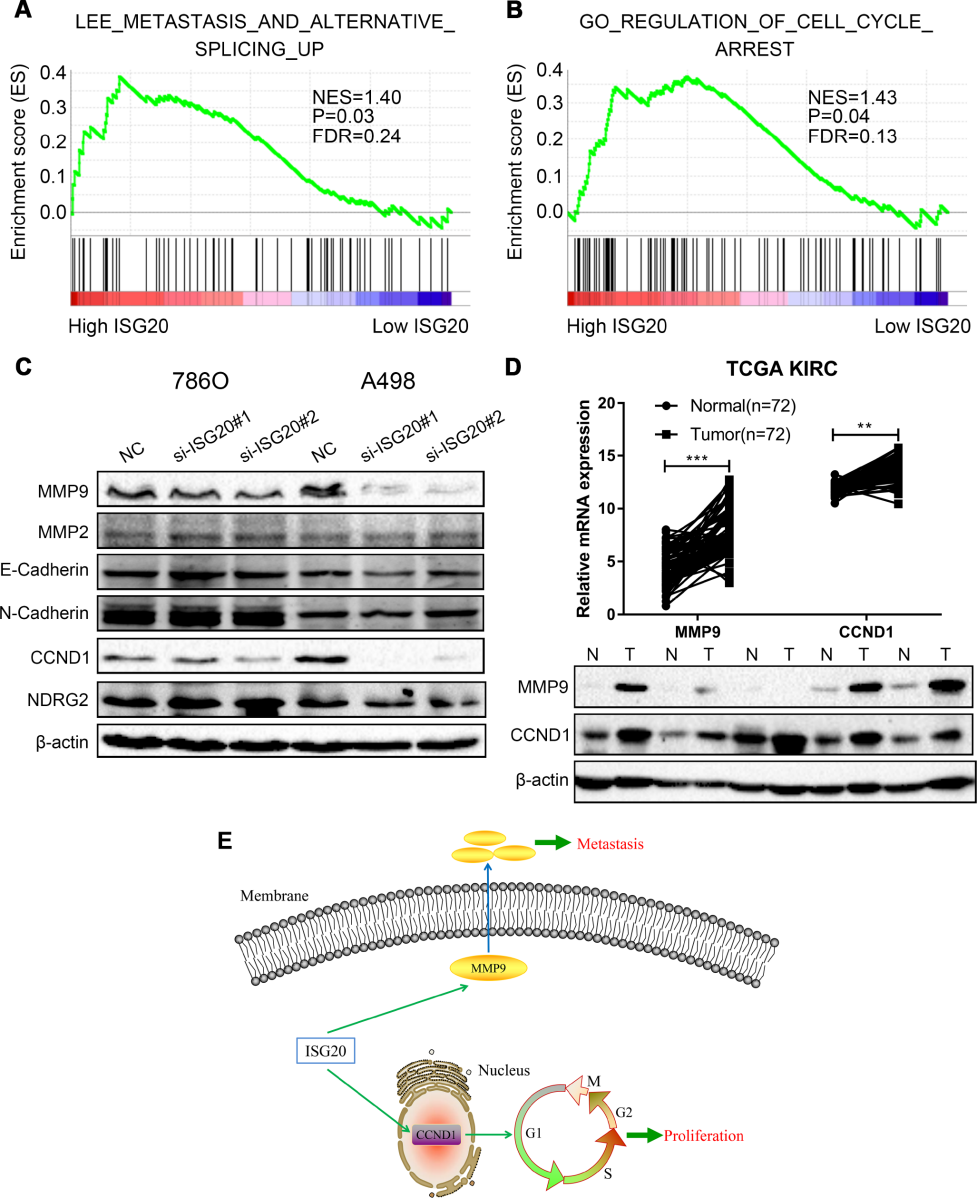
**ISG20 positively regulated the expression of MMP9/CCND1 in ccRCC.** (**A**–**B**) GSEA revealed that the high ISG20 expression group was associated with metastasis pathways and cell cycle pathways. (**C**) The expression of MMP9 and CCND1 were down-regulated in ccRCC cells with knockdown of ISG20. (**D**) The expression of MMP9 and CCND1 were elevated in the TCGA KIRC dataset and ccRCC tissues. (**E**) The schematic representation of elevated the expression of ISG20 could drive tumor progression via up-regulating the expression of MMP9/CCND1. ccRCC: clear cell renal cell carcinoma; TCGA KIRC: The Cancer Genome Atlas Kidney Clear Cell Carcinoma. Data are represented as mean ± SD. ***, P < 0.001; **, P < 0.01; *, P < 0.05.

GSEA results indicated that the high expression of the ISG20 group mainly enriched in metastasis pathways and cell cycle pathways (Figure 8A, 8B). We also found that the silencing of ISG20 could significantly down-regulate the expression of MMP9 and CCND1 in ccRCC (Figure 8C). In addition, both MMP9 and CCND1 were both up-regulated in ccRCC (Figure 8D). Therefore, we believed that ISG20 might promote the progression of ccRCC through up-regulating CCND1 and MMP9 (Figure 8E).

## DISCUSSION

ccRCC is one of the most common neoplasms characterized by high metastasis and recurrence rates. At the time of diagnosis, with about 30% of patients exist metastatic lesions due to lack of effective biomarkers and obvious clinical manifestations [8]. Molecular targeted therapy prolongs the survival time of some patients, but there are still many patients who are insensitive to targeted therapy [26, 27]. At present, immunotherapy, specifically immune checkpoint inhibitors, has become a new promising strategy for ccRCC [28–30]. However, intrinsic resistance or acquired resistance of immunotherapy is still observed [31]. Therefore, it is very necessary to further study the molecular mechanisms of progression and discover new therapeutic targets of ccRCC.

In this present study, we performed differential analysis and functional annotation to screen important genes in ccRCC. WGCNA and Cox regression analysis were used to reduce dimension and identify hub genes. ISG20, HJURP, and FOXM1 were selected as candidate biomarkers for further analysis and study.

GO enrichment analysis indicated that the biological functions of DEGs were mainly correlated to transmembrane transporter activity and cell-cell adhesion. Numerous studies have shown that transmembrane transport plays an important role in various physiological responses and functions [32–34]. For tumor cells, abnormal transmembrane transport may be a vital factor in maintaining their survival and malignancy [35, 36]. It has been reported that monocarboxylate transporter 1 (MCT1) and monocarboxylate transporter 4 (MCT4) promotes proliferation and metastasis of renal cancer cells [37]. In hepatocellular carcinoma, previous study proved that YAP1/TAZ activated the mTORC1 pathway via up- regulating amino acid transporters (SLC38A1 and SLC7A5) to promote cell growth [38]. Cell adhesion is another important biological behavior which obviously associated with cell proliferation, migration, and invasion [39], especially in cancers. Labernadie et al. uncovered that cancer-associated fibroblasts (CAFs) enhanced tumor invasion via heterophilic adhesion between CAFs and tumor cells [40]. In the glioblastoma, acetyl-coenzyme A (acetyl-CoA) acted as an activator for the Ca^2+^-NFAT signaling pathway to drive cell adhesion and migration [41]. In our present study, KEGG analysis also showed that the DEGs were enriched in cell adhesion molecules (CAMs) pathway. Furthermore, KEGG enrichment analysis revealed that the DEGs were obviously associated with multiple metabolic pathways. ccRCC is known for metabolic disorders. Recent studies indicated that abnormal metabolism played an important role in the occurrence and progression of ccRCC. Bianchi et al. proved that aerobic glycolysis was a grade-dependent feature and fatty acid oxidation was needed in different grade tumors [42]. Multi-omics characterization also revealed that abnormal glycolysis and pentose phosphate pathway promoted tumor growth [43]. Lucarelli et al. indicated that aerobic glycolysis reprogramming is pivotal in the initial phases of tumorigenesis in ccRCC [44]. Therefore, a better understanding of the molecular mechanisms underlying this metabolic alteration will be crucial for the identification of novel potential therapeutic targets and biomarkers.

It was worth mentioning that the DEGs were significantly associated with the peroxisome proliferators-activated receptors (PPAR) signaling pathway. A large number of studies have demonstrated that the occurrence and development of tumors are closely related to PPAR signaling pathways. It has been reported that PPAR-delta maintains cell survival in an energy-poor environment for chronic lymphocytic leukemia [45]. Zuo et al. also verified that up-regulated PPAR-δ/β could promote the susceptibility to colon cancer in villin-PPAR mice models [46]. Thus, in-depth study of the mechanisms of the PPAR pathway will help us in conquering cancer.

In this study, we selected ISG20 for further study through the validation of ccRCC tissues. ISG20, an interferon regulated gene, codes for a 20-kDa protein with 181-amino acid [47]. Both type I (IFN-α/β) and type II (IFN-γ) IFNs can induce the expression of ISG20 [17]. As a 3'-5' exonuclease, ISG20 cleaves both DNA and RNA to protect against virus and bacteria [48]. Qu et al. found that ISG20 reduced influenza A virus (IAV) replication by interacting with nucleoprotein [49]. Apart from fighting against pathogens, ISG20 also plays an important role in other diseases [50], especially in tumors. It has been reported that ISG20 is up-regulated in cervical cancer [51]. In this study, we also found that the expression of ISG20 was elevated in online databases (TCGA and Oncomine) and ccRCC samples. In human glioma, ISG20 was positively correlated to immune checkpoints (PD-1 and PD-L1) and suppressed the adaptive immune response. In addition, high expression of ISG20 predicted poor overall survival in glioma [52]. Similarly, patients with high expression of ISG20 had shorter overall survival time in our present study. We found that ISG20 was positively associated with the clinical stage (TNM stage and Grade stage) in ccRCC. Furthermore, ROC curve analysis indicated that ISG20 could effectively differentiate ccRCC samples from normal samples. These results suggested that ISG20 might serve as a potential biomarker in ccRCC.

To further reveal the biological function and molecular mechanisms of ISG20, GSEA was performed and indicated that high expression of ISG20 mainly enriched in metastasis and cell cycle pathways. Functional experiments also verified that knockdown of ISG20 could obviously inhibit proliferation, migration, and invasion of ccRCC cells. In addition, ISG20 could positively regulate the expression of MMP9 and CCND1 in ccRCC cells in our study. MMP9 (matrix metalloproteinase 9), a member of the matrix metalloproteinase (MMP) family, is involved in the breakdown of extracellular matrix [53]. Type IV and V collagens are the major substrates for MMP9. A large number of studies have proved that MMP9 plays an important role in tumorigenesis, proliferation, apoptosis, invasion, and angiogenesis as well [54–57]. Moreover, it has been reported that the expression and activation of MMP9 are regulated by multiple signal pathways, such as STAT3 pathway [58, 59], JNK pathway [60, 61] and PI3K/AKT pathway [62]. A number of studies also uncovered that MMP9 could promote the migration and invasion of ccRCC cells [63, 64]. Gong et al. verified that P2RX6 facilitated the invasion and metastasis of RCC cells through the Ca^2+^-p-ERK1/2-MMP9 signal pathway [65]. CCND1 (cyclin D1), a member of the highly conserved cyclin family, acts as a regulator of CDK kinases for mediating cell cycle [66, 67]. Mutations, amplification and overexpression of CCND1 are observed frequently in a variety of tumors and these may contribute to tumorigenesis [68–70]. Several studies indicated that non-coding RNA was involved in tumor progression via regulating the expression of CCND1. Ai et al. revealed that LINC01355 inhibited cell proliferation through suppressing the transcription of CCND1 [71]. In cervical cancer, a research group demonstrated that miR-2861 suppressed tumor cell growth and invasion by targeting EGFR/AKT2/CCND1 pathway [72]. However, the specific molecular mechanisms between ISG20 and MMP9/CCND1 are still unclear and need further study.

In summary, ISG20, HJURP, and FOXM1 were identified as hub genes via WGCNA and Cox regression analysis in this study. Clinical samples analysis showed that only ISG20 had an obvious difference between ccRCC tissues and normal renal tissues. Survival analysis and ROC curve analysis indicated that ISG20 had good diagnostic and prognostic value, which could become a candidate biomarker in ccRCC. In addition, high expression of ISG20 promoted the proliferation, migration, and invasion of ccRCC cells via regulating MMP9/CCND1 expression. To the best of our knowledge, this is the first study on the biological functions of ISG20 in ccRCC. These findings may provide a new therapeutic target for ccRCC. However, the specific mechanisms of ISG20 in ccRCC still need further research.

## MATERIALS AND METHODS

### Data download and study design

**Figure 9:**
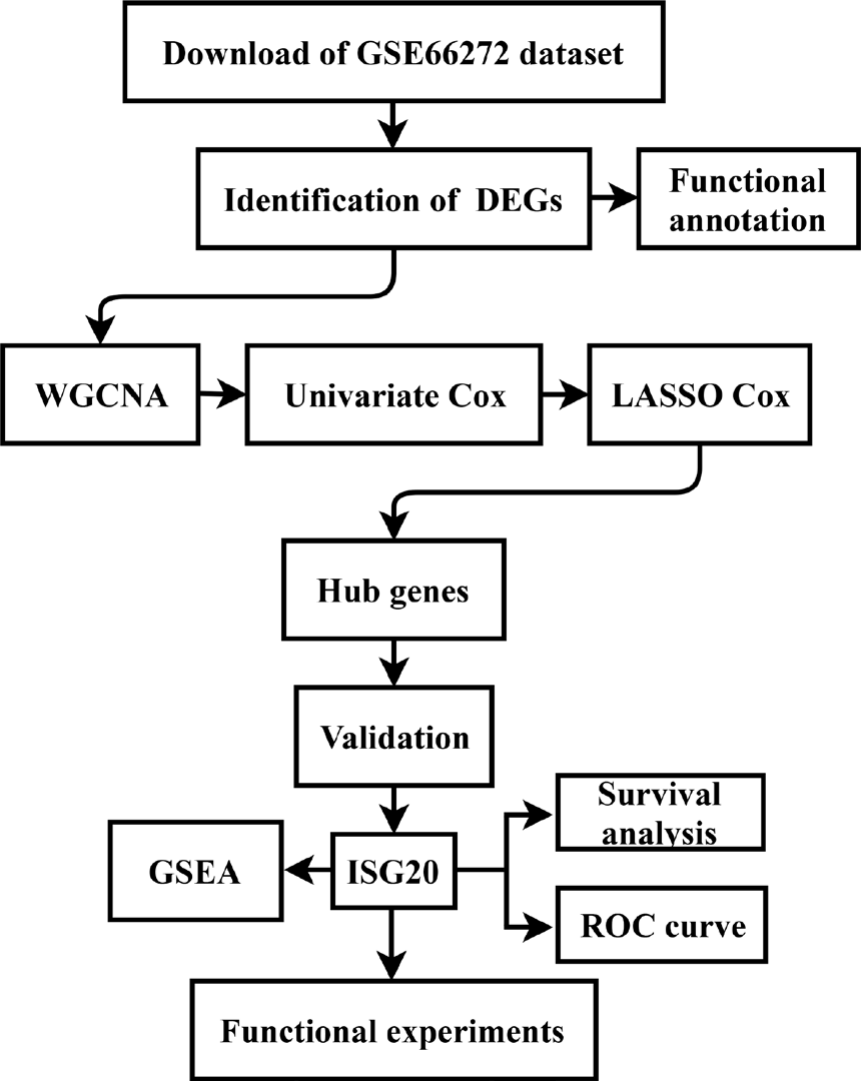
**Flow diagram of the study. Data collection and analysis were exhibited in the flow diagram.** DEGs: differentially expressed genes; WGCNA: weighted gene co-expression network analysis; LASSO: least absolute shrinkage and selection operator; GSEA: gene set enrichment analysis; ROC: receiver operator characteristic.

The gene expression data and clinical data were downloaded from the GSE66272 dataset and The Cancer Genome Atlas (TCGA) database (https://www.cancer.gov/tcga). The microarray data of GSE66272 included 52 matched ccRCC tissues and adjacent normal tissues. Furthermore, the study design was exhibited in a flow diagram (Figure 9).

### Identification of differentially expressed genes (DEGs)

The “limma” package [73] was used to screen DEGs according to the cutoff criterion of adjusted P-value (adj. P) < 0.05 and |log Fold Change| (|log FC|) > 2.0. A heat map was drawn to exhibit the expression difference between ccRCC samples and normal samples of the top 50 DEGs.

### Functional and pathway enrichment analysis

Gene Ontology (GO) analysis and Kyoto Encyclopedia of Genes and Genomes (KEGG) pathway analysis were performed to further study the potential biological function of DEGs via the “clusterProfiler” package [74] in R. P value < 0.05 was selected as the cutoff point.

### Weighted gene co-expression network analysis (WGCNA)

The “WGCNA” package [75] was used to construct a co-expression network in R. Firstly, GSE66272 was evaluated via sample clustering to detect outliers. No samples were removed according to the height cutoff point = 60 in this study (Figure 1A). Then, the Pearson’s correlation matrices were performed between each of the gene pairs. A weighted adjacency matrix was constructed using a power function a_mn_ = |c_mn_|^β^ (c_mn_ = Pearson’s correlation between gene m and gene n; a_mn_ = adjacency between gene m and gene n). The adjacencies achieved scale-free topology based on the soft threshold power β. Next, the adjacencies were transformed into a topological overlap matrix (TOM). Average linkage hierarchical clustering was performed to divide DEGs into different modules according to the correlations between each gene. Genes with high absolute correlation were clustered into the same module. Finally, the correlation between module eigengenes (MEs) and clinical traits was calculated to identify the clinically significant modules. Furthermore, we calculated the correlation between genes and clinical traits (cor.geneTraitSignificance) and the correlation between genes and MEs (cor.geneModuleMembership) as well. In this study, we selected |cor.geneTraitSignificance| > 0.4 and |cor.geneModuleMembership| > 0.8 of T stage, M stage and Grade as the cutoff criterion to screen clinical key genes.

### Cox regression analysis

Firstly, the “survival” package (https://CRAN.R-project.org/package=survival) was applied to perform univariate cox regression analysis for both overall survival (OS) and disease-free survival (DFS) in R. Genes with p value < 0.05 were identified as useful genes. Then, least absolute shrinkage and selection operator (LASSO) cox regression analysis was performed to further screen hub genes via “glmnet” [76, 77] package in R.

### Hub genes validation in TCGA, GEO, and Oncomine databases

The mRNA expression data of hub genes were downloaded from The Cancer Genome Atlas (TCGA), Gene Expression Omnibus (GEO) and Oncomine (https://www.oncomine.org) databases including Beroukhim Renal, Gumz Renal, Jones Renal, and Yusenko Renal dataset. GraphPad Prism software was used to verify the difference between ccRCC samples and normal samples. P value < 0.05 was considered statistically significant.

### Survival analysis and receiver operator characteristic (ROC) curve analysis

Survival data were obtained from the TCGA database. The ccRCC samples were divided into high expression group and low expression group according to the median mRNA expression of hub genes. Kaplan-Meier survival curves of overall survival (OS) and disease-free survival (DFS) were performed by GraphPad Prism. Meanwhile, the ROC curves were also drawn by GraphPad Prism to evaluate the diagnostic value of hub genes. P value < 0.05 was considered statistically significant.

### Gene set enrichment analysis (GSEA)

All ccRCC samples were divided into high expression group and low expression group according to the median mRNA expression of hub genes. Then, GSEA software [78, 79] was used to find potential molecular mechanisms of hub genes. Nominal P < 0.05 and false discovery rate (FDR) < 0.25 were selected as cutoff criteria.

### Human ccRCC specimens

A total of 37 pairs of ccRCC tissues and adjacent normal renal tissues (5 cm away from the margin of the tumor tissues) were collected from Wuhan Union Hospital between 2017 and 2018. All patients were provided with informed consent. Moreover, this study was approved by the Human Research Ethics Committee of Huazhong University of Science and Technology (HUST).

### Cell culture

The 786O, Caki-1, A498, OSRC-2, and HK-2 cell lines were purchased from the American Type Culture Collection (ATCC) in this study. All cells were cultured in Dulbecco's modified Eagle's medium (Gibco, USA) with 10% fetal bovine serum (FBS) and 1% penicillin-streptomycin (Gibco, USA). Moreover, all cells were maintained in 37 °C and 5% CO_2_ culture environment.

### RNA extraction and quantitative real-time PCR (qRT-PCR)

Total RNA of cells and tissues was extracted with the TRIzol reagent (Invitrogen, CA). Then, the concentration and purity of RNA were detected via using Nanodrop 2000c spectrophotometer (Thermo Scientific, USA). Reverse transcription kit (Takara, China) was used to synthesize cDNA for further analysis. The qRT-PCR assay was performed to amplify cDNA with SYBR Green Master Mix (Vazyme, China). Primer sequences were listed as follows: GAPDH Forward: 5′- GCACCGTCAAGGCTGAGAAC-3′; GAPDH Reverse: 5′- TGGTGAAGACGCCAGTGGA-3′; ISG20 Forward: 5′- CTCGTTGCAGCCTCGTGAA-3′; ISG20 Reverse: 5′- CGGGTTCTGTAATCGGT GATCTC-3′; HJURP Forward: 5′- CACAAAGC CATCAAGCATCATC-3′; HJURP Reverse: 5′- TCA GAGCAGGGTATGAAGTTCT-3’; FOXM1 Forward: 5′- CGTCGGCCACTGATTCTCAAA-3′; FOXM1 Reverse: 5′- GGCAGGGGATCTCTTAGGTTC-3′.

### Western blotting (WB)

Western blotting was performed as previously described [80]. In brief, the protein of cells and tissues were extracted with the radio-immunoprecipitation assay (RIPA) lysis buffer (Beyotime, China) containing 1mM Phenylmethylsulfonyl fluoride (Beyotime, China) and 1X protease inhibitor cocktail (MCE, USA). The concentration of protein was detected using the BCA assay kit (Beyotime, China). 60ug of protein was subjected to 12% SDS-PAGE and transferred to PVDF membranes (Millipore, USA). Then, the membranes were blocked in 5% nonfat dried skimmed milk for 2h at room temperature. After that, the PVDF membranes were incubated with primary antibodies containing ISG20 (1:100, Abclonal, China, A14744), GAPDH (1:50000, Abclonal, China, AC002), β-actin (1:50000, Abclonal, China, AC026), MMP9 (1:1000, Abclonal, China, A2095), CCND1 (1:10000, Abcam, ab134175) and NDRG2 (1:5000, Abcam, ab169775) overnight at 4 °C. Finally, the membranes were incubated with corresponding secondary antibodies (1:3000, Proteintech, China, SA00001-1 and SA00001-2) for 1.5h at room temperature and visualized with the ChemiDoc-XRS+ system (Bio-Rad, USA).

### Immunohistochemical (IHC) assay

IHC assay was performed as previously described [81]. In short, the ccRCC tissues and adjacent renal tissues were fixed using the following step: formalin fixation, dehydration, and paraffin embedding. After that, the tissue sections were incubated with a primary antibody against ISG20 (1:50, Abclonal, China, A14744) overnight at 4 °C. Then, the tissue sections were washed with PBS two times and incubated with a second antibody (1:400, Proteintech, China, SA00001-2) for 1.5h at room temperature.

### Transient transfection assay

The siRNA oligonucleotide sequences targeting ISG20 (si-ISG20) and the negative control (si-NC) siRNA were designed and synthesized from TranSheepBio (Shanghai, China). The si-ISG20 and si-NC with a final concentration of 75 nM were transfected into the ccRCC cells of 70% confluence with Lipofectamine® 3000 (Invitrogen, USA) according to the manufacturer's protocol. Cells were collected 48h after si-RNA transfection as subsequent assays. The si-ISG20 sequences were listed as below: si-ISG20#1: 5′-GAGAUCACCGAUUACAGAATT-3′; si-ISG20#2: 5′-GAUCCUGCAGCUCCUGAAATT-3′.

### Cell viability assays

In this study, cell counting kit-8 (CCK-8) assay and colony formation assay were performed to evaluate the effect of ISG20 on cell proliferation.

Cell counting kit-8 assay: 1000 cells were planted in 96-well plates per well with 100ul of medium. Then, CCK-8 solution (MCE, USA) was added to 96-well plates with 10ul per well. After incubation for 2.5h at 37 °C, the optical density (OD) value of each well was measured at 450 nm with a spectrophotometer (Bio-Rad, USA). The OD value was assessed at after 0, 24, 48, 72 and 96h upon treatments, respectively.

Colony formation assay: 1000 cells were seeded in 6-well plates per well with 2ml complete medium and cultured for 2 weeks. Then, the cells were fixed with methanol and stained with 0.1% crystal violet.

### Transwell assay

Firstly, cells were incubated in FBS-free medium for 24h. Secondly, a total of 10000 cells were seeded into the upper chamber with 200 μl of FBS-free medium for the migration assay and 20000 cells were seeded into the upper chamber which was pre-coated with Matrigel (Bio-Rad, USA) for the invasion assay. In addition, 600ul medium with 10%FBS was added into the bottom chamber. Thirdly, the invasive cells were fixed with methanol and stained with 0.1% crystal violet after incubated for 24h. Finally, five fields were randomly selected to count the cells.

### Wound healing assay

Cells were seeded into the 6-well plates with equal numbers. Then, cells were wounded by a 10ul pipette tip when its confluence reached 80-90%. Images of the wound were obtained at 0 and 24h.

### Statistical analysis

All statistical analyses were performed using GraphPad Prism 7.0 (GraphPad software, Inc., La Jolla, CA, USA) and each experiment was conducted in triplicate. All data were represented as mean ± SD. Student’s t-test and Pearson’s χ^2^ test were used to analyze the data in this study. The significance value was determined when p < 0.05.
